# OA10.01. The Yoga Dosing Study: comparing once vs. twice per week yoga classes for chronic low back pain in predominantly low income minority populations

**DOI:** 10.1186/1472-6882-12-S1-O37

**Published:** 2012-06-12

**Authors:** R Saper, A Boah, J Keosaian, J Weinberg, K Sherman

**Affiliations:** 1 Boston University School of Medicine, Boston, USA; 2 Boston University School of Public Health, Boston, USA; 3 Group Health Research Institute, Seattle, USA

## Purpose

Previous studies suggest yoga is effective for mild to moderate chronic low back pain (CLBP) in mostly white higher socioeconomic status (SES) populations. However, little is known regarding yoga’s optimal dose or its effectiveness for more severe CLBP in diverse lower SES populations.

## Methods

From September-December 2011, we conducted a 12-week RCT comparing once vs. twice-weekly standardized 75-minute hatha yoga classes for 95 adults with nonspecific CLBP. Recruitment and classes occurred in a large safety-net hospital and five affiliated community health centers in Boston, Massachusetts. Primary outcomes were mean low back pain intensity in the previous week (0-10) and back-related function (Modified Roland-Morris Disability, MRMD, 0-23). We used two-sample t-tests to compare once/week vs. twice/week mean change scores (baseline-12 weeks) for pain and MRMD. Analyses used the intention-to-treat principle.

## Results

Participants were on average 48 years old, 76% female, 82% non-white, 63% with annual household incomes <$30,000, and 35% with high school education or less. Baseline pain intensity (6.9, SD 1.6) and MRMD (13.7, SD 5.0) were consistent with moderate-severe CLBP. Baseline characteristics of the once/week (n=49) and twice/week (n=46) groups were similar. Overall class attendance was 73% and 62% for the once/week and twice/week participants, respectively. Both groups practiced yoga at home on average 3-4 days/week. Each group experienced statistically significant (p<.0001) and clinically meaningful improvements in pain and function: Mean pain change scores for the once/week and twice/week groups were -2.1 (SD 2.7) and -2.4 (SD 2.2), respectively. Mean MRMD change scores for the once/week and twice/week groups were -5.2 (SD 6.5) and -4.9 (SD 4.4), respectively. There were no statistically significant differences between the two groups for pain or MRMD.

## Conclusion

Twelve weeks of either once or twice-weekly hatha yoga classes augmented by home practice were similarly effective for moderate to severe CLBP in a diverse predominantly lower SES population.

